# Integrating physical activity into esports practice: predicting mental well-being and cognitive fatigue through a dual SEM-XAI approach

**DOI:** 10.3389/fpsyg.2026.1831947

**Published:** 2026-07-13

**Authors:** Xiaoming Zhou, Renjie Ma

**Affiliations:** 1 Department of Physical Education, Chang’an University, Xi’an, China; 2 School of Journalism and Communication, Beijing Sport University, Beijing, China; 3 School of Marxism, Zhengzhou University, Zhengzhou, China

**Keywords:** biopsychosocial model, esports, explainable machine learning, mental well-being, physical activity, structural equation modeling

## Abstract

**Objective:**

The rapid professionalization of electronic sports (esports) has introduced distinct occupational health challenges, characterized by prolonged sedentary behavior, severe sleep disruptions, and high cognitive fatigue. While existing literature frequently focuses on the psychopathology of gaming disorder, the protective role of physical activity (PA) in ameliorating these specific hazards remains under-investigated. Grounded in the Biopsychosocial Model, this study aimed to elucidate the underlying mechanisms and identify the non-linear dose–response patterns through which PA is associated with esports players’ mental well-being.

**Methods:**

A cross-sectional survey was conducted among 512 collegiate and semi-professional esports practitioners. The study employed an innovative dual-analytical approach: Structural Equation Modeling (SEM) was utilized to test the serial mediating roles of sleep quality and cognitive fatigue, alongside the moderating effect of esports engagement. Concurrently, an Explainable Artificial Intelligence (XAI) framework—specifically Random Forest combined with SHapley Additive exPlanations (SHAP)—was applied to uncover complex, non-linear predictive patterns and behavioral thresholds.

**Results:**

SEM results indicated that PA positively predicted mental well-being, a relationship serially mediated by improved sleep quality and reduced cognitive fatigue. Furthermore, esports engagement intensity significantly moderated this pathway, suggesting that highly engaged players derived more profound protective benefits from PA. Crucially, the SHAP analysis revealed a robust non-linear pattern: accumulating approximately 600–1,200 MET-min/week showed the steepest positive predictive contribution to mental well-being, with signs of plateauing beyond approximately 1,800 MET-min/week. Beyond this zone, the predicted psychological benefits reached a plateau, indicating diminishing returns.

**Conclusion:**

Physical activity is robustly associated with cognitive resilience and mental health outcomes in esports practitioners, suggesting its role as a potential biological regulatory factor potentially relevant to sustaining well-being in highly sedentary digital arenas. By integrating SEM with Explainable AI, this study offers preliminary, hypothesis-generating evidence that may inform future refinement of general PA guidelines for the esports phenotype, and suggests a possible direction for developing precision-based, Just-In-Time Adaptive Interventions (JITAI) to safeguard occupational well-being in the digital age.

## 1 Introduction

The global proliferation of electronic sports (esports) has fundamentally reshaped the landscape of competitive activities. Recognized globally as a legitimate and highly competitive domain, esports demands extraordinary cognitive agility, rapid decision-making, and sustained visual vigilance from its practitioners (Campbell et al., 2018). The transition of gaming from a recreational pastime into highly professionalized digital arenas has precipitated a unique occupational health paradox. Unlike traditional athletes who undergo rigorous physical conditioning as an integral component of their sport, esports players are typically subjected to prolonged periods of sedentary behavior intertwined with acute psychological stress (DiFrancisco-Donoghue et al., 2019). A growing body of empirical literature has begun to document the physical and psychological detriments associated with extensive esports participation, including musculoskeletal disorders, disrupted circadian rhythms, and profound cognitive fatigue (Yin et al., 2020). Despite these recognized occupational risks, mainstream health psychology research has disproportionately focused on the psychopathology of excessive gaming, often conflating high-level esports engagement with gaming disorders. This approach largely neglects the exploration of protective, proactive health behaviors that could mitigate adverse outcomes.

To address this critical gap, the present study investigates the associational role of physical activity in preserving and enhancing the mental well-being of esports practitioners. Drawing upon the Biopsychosocial Model (Engel, 1977), which posits that health outcomes result from complex biological, psychological, and social interactions, this research conceptualizes physical activity as a systemic psychosocial intervention capable of counteracting the occupational hazards specific to esports. Current empirical investigations into physical activity within esports contexts are relatively nascent and are often constrained by traditional linear methodological paradigms (Trotter et al., 2020). While linear models provide valuable insights into broad correlational associations, they frequently fail to capture the complex, non-linear dose–response relationships inherent in human health behaviors.

Consequently, this study adopts a rigorous dual-analytical framework integrating Structural Equation Modeling (SEM) and Explainable Machine Learning (XAI). SEM is utilized to test theory-driven, structural pathways, specifically examining how physical activity is associated with mental well-being through the serial mediation of sleep quality and cognitive fatigue, and how this mechanism is contingent upon the intensity of esports engagement. Concurrently, advanced machine learning techniques, specifically the Random Forest algorithm coupled with SHapley Additive exPlanations (SHAP) (Lundberg et al., 2020), are employed to transcend the limitations of linear assumptions. By utilizing XAI, this research aims to identify the precise, non-linear patterns of physical activity that are predictively associated with optimal mental well-being outcomes. Ultimately, this study seeks to integrate health psychology into the highly digital and sedentary context of esports practice, providing associational, data-driven parameters to inform future health health strategies and organizational guidelines.

## 2 Theoretical framework and hypotheses

### 2.1 The biopsychosocial paradigm in esports contexts

The Biopsychosocial Model provides a comprehensive theoretical framework for understanding health dynamics in non-traditional, technology-driven environments. In the context of esports, the biological domain encompasses the physiological toll of extended sedentary screen time and the restorative physiological cascades triggered by physical exertion, such as the regulation of cortisol levels and the release of brain-derived neurotrophic factors (Biddle et al., 2021). The psychological domain involves the cognitive exhaustion and emotional dysregulation stemming from high-stakes digital competition. The social domain relates to the intensive training regimes and the broader, often isolating, digital culture. Within this paradigm, physical activity functions as a systemic intervention that simultaneously addresses biological deficits and psychological distress, fostering holistic well-being rather than merely treating localized symptoms (Reigal et al., 2020).

### 2.2 Physical activity, sleep quality, cognitive fatigue, and mental well-being

Extensive evidence from traditional sports and occupational health psychology demonstrates that regular moderate-to-vigorous physical activity is a robust predictor of mental well-being (Pereira et al., 2022). For esports players, who frequently suffer from prolonged sleep latency and disrupted rapid eye movement cycles due to late-night screen exposure, physical activity may serve as a potent thermoregulatory and circadian reset mechanism (Kemp et al., 2021). Improved sleep architecture is inexorably linked to cognitive restoration. Esports requires relentless selective attention and executive functioning, leading inevitably to severe cognitive fatigue (Toth et al., 2020). Sleep acts as the primary physiological mechanism for clearing metabolic waste from the brain and facilitating cognitive recovery. It is therefore postulated that physical activity is associated with enhanced sleep quality, which in turn may attenuate cognitive fatigue, ultimately contributing to heightened mental well-being. Furthermore, the associational strength between physical activity and well-being outcomes is likely contingent upon the intensity of esports engagement. Individuals with higher levels of esports engagement experience more pronounced sedentary and cognitive burdens. Thus, they may benefit disproportionately from the compensatory neurobiological mechanisms of physical activity. Based on these theoretical delineations, the following hypotheses were formulated:

*Hypothesis 1 (H1)*: Physical activity is positively associated with mental well-being among esports practitioners.

*Hypothesis 2 (H2)*: Sleep quality and cognitive fatigue serially mediate the relationship between physical activity and mental well-being.

*Hypothesis 3 (H3)*: Esports engagement intensity moderates the mediated pathways, such that the association between physical activity and cognitive fatigue reduction is stronger for individuals with higher engagement levels.

### 2.3 The imperative for explainable machine learning

While SEM is highly effective in validating theoretical constructs and directional hypotheses, it inherently assumes linearity within its structural paths (Anderson and Gerbing, 1988). Exercise physiology and health psychology literature increasingly recognize that the relationship between physical activity and health outcomes is often asymptotic or non-linear. To uncover the precise critical predictive pattern at which esports players show maximum psychological benefit before experiencing diminishing returns, this study integrates a Random Forest algorithm interpreted via SHAP values (Breiman, 2001; Lundberg et al., 2020). This XAI approach not only robustly predicts mental well-being but also mathematically unpacks the machine learning model, providing exploratory, hypothesis-generating dosage patterns that may inform future clinical health psychology health strategies.

## 3 Methods

### 3.1 Participants and procedure

A cross-sectional survey design was employed for this investigation. Participants were recruited from various collegiate esports programs and semi-professional gaming clubs across major metropolitan areas in China using purposive and snowball sampling techniques (Schinka and Velicer, 2003). Inclusion criteria required participants to be actively involved in competitive esports training or competition, playing a minimum of 15 h per week. Data were collected via an encrypted online survey platform. Informed consent was obtained from all participants prior to data collection, and the study protocol was approved by the Institutional Review Board of the author’s affiliated university.

Data screening procedures were implemented to ensure response quality. Responses with more than 10% missing data on any single scale were first excluded (*n* = 23). For the remaining cases, Little’s MCAR test indicated that missing data were missing completely at random (*χ*^2^(47) = 51.23, *p* = 0.31), and the overall missing data rate was low (<2%). Pairwise deletion was applied for the correlation matrix, and Full Information Maximum Likelihood (FIML) was used within the SEM framework to handle the minimal remaining missingness without further case exclusion. Subsequently, multivariate outliers were identified using Mahalanobis distance (D^2^) with a conservative threshold of *p* < 0.001 (critical χ^2^ value = 24.32 with 7 degrees of freedom corresponding to the variables included in the outlier screening procedure), resulting in the removal of 17 cases. After these screening steps, the final analytical sample comprised 512 participants (Male = 453, Female = 59). This gender ratio reflects current demographic realities within competitive esports domains (Bányai et al., 2019). The mean age of the participants was 21.45 years (Standard Deviation = 2.87). Participants reported engaging in esports for an average of 28.50 h per week (Standard Deviation = 8.35).

### 3.2 Measures

All constructs were assessed using standardized, open-access psychometric instruments.

#### 3.2.1 Physical activity

The short form of the International Physical Activity Questionnaire (IPAQ-SF) was utilized to assess physical activity levels (Craig et al., 2003). Participants reported the frequency and duration of vigorous, moderate, and walking activities over the past 7 days. Data were transformed into Metabolic Equivalent of Task (MET) minutes per week following standard IPAQ scoring protocols, providing a continuous measure of overall energy expenditure.

#### 3.2.2 Sleep quality

Sleep quality was evaluated using the global score of the Pittsburgh Sleep Quality Index (PSQI) (Buysse et al., 1989). The instrument assesses seven components of sleep, including subjective quality, latency, duration, and disturbances. Scores range from 0 to 21, with higher scores indicating poorer sleep quality. For analytical congruency within the SEM model, the PSQI scores were reverse-coded prior to analysis. The internal consistency in the current study was adequate (Cronbach’s alpha = 0.84).

#### 3.2.3 Cognitive fatigue

Cognitive fatigue was measured using the personal and work-related fatigue subscales of the Copenhagen Burnout Inventory (CBI) (Kristensen et al., 2005). Adapted for the esports context, participants responded to 13 items on a 5-point Likert scale. Higher scores reflect greater cognitive and occupational fatigue (Cronbach’s alpha = 0.89).

#### 3.2.4 Mental well-being

The 5-item World Health Organization Well-Being Index (WHO-5) served as the primary dependent variable (Topp et al., 2015). This unidimensional scale measures subjective psychological well-being over the past 2 weeks on a 6-point scale. The WHO-5 is highly regarded for its clinical validity (Cronbach’s alpha = 0.91).

#### 3.2.5 Esports engagement intensity

The 9-item Internet Gaming Disorder Scale-Short Form (IGDS9-SF) was utilized as a continuous metric of engagement intensity and psychological absorption in gaming activity (Pontes and Griffiths, 2015). Items were scored on a 5-point scale (Cronbach’s alpha = 0.87). It is important to acknowledge that the IGDS9-SF was originally developed and validated as a screening instrument for Internet Gaming Disorder symptoms. In the present study, it was conceptually repurposed as a continuous proxy for esports engagement intensity, operationalizing the degree of psychological and behavioral absorption in esports activity. This operationalization is theoretically defensible for the following reasons. First, within a non-clinical, semi-professional esports sample—where gaming constitutes a legitimate occupational activity rather than a pathological behavior—elevated IGDS9-SF scores are more parsimoniously interpreted as reflecting high occupational investment and behavioral absorption rather than disordered gaming per se. Second, the item content of the IGDS9-SF (e.g., preoccupation with gaming, tolerance for gaming duration, difficulty reducing gaming, functional salience of gaming) captures the intensity of gaming involvement and its centrality to the individual’s lifestyle, both of which are theoretically relevant moderators of the PA–well-being relationship. Third, in the current sample, the mean IGDS9-SF score (M = 22.30, SD = 6.85) falls well below the clinical cutoff suggested for probable problematic gaming, indicating that the observed variance primarily reflects subclinical engagement intensity gradations rather than clinical pathological symptoms. Nevertheless, we acknowledge that future research should prioritize purpose-built engagement intensity instruments to more cleanly separate occupational involvement from disordered gaming constructs, and the present moderation findings should be interpreted with this psychometric caveat in mind.

### 3.3 Data analysis strategy

The analytical procedure involved two distinct phases.

First, SEM was executed using the lavaan package in R (Rosseel, 2012). The SEM was specified as a reflective measurement model with four latent variables: Sleep Quality (7 items from PSQI), Cognitive Fatigue (13 items from CBI), Esports Engagement (9 items from IGDS9-SF), and Mental Well-Being (5 items from WHO-5). Physical Activity (MET-minutes/week) was modeled as a single observed (manifest) variable, consistent with the continuous composite scoring protocol of the IPAQ-SF. Item parceling was applied to the multi-item latent variables to reduce model complexity and improve the indicator-to-sample-size ratio, and the parceled indicators were used in the CFA and SEM analyses. Prior to full SEM estimation, multivariate normality was assessed using Mardia’s test, which revealed non-trivial excess kurtosis in the data (Mardia’s kurtosis = 18.42, *p* < 0.001). Accordingly, the structural model was estimated using Maximum Likelihood with Satterthwaite-corrected robust standard errors (MLR estimator in lavaan), which provides asymptotically correct standard errors and fit statistics under non-normality. The moderation term (Engagement × PA interaction) was specified using the unconstrained product indicator approach, with mean-centering applied to both focal variables prior to interaction term creation to reduce multicollinearity. Age, gender, and weekly esports hours were included as covariates in all structural paths leading to the outcome variable. Degrees of freedom for the measurement model [df = 142] were derived from the standard formula: [p(p + 1)/2] − q, where p = number of observed indicators and q = number of freely estimated parameters. A Confirmatory Factor Analysis (CFA) was conducted to establish the measurement model’s validity. Mediation effects were tested utilizing 5,000 bootstrap resamples to generate 95% bias-corrected confidence intervals (Hayes, 2018). Model fit was evaluated based on established criteria, including the Comparative Fit Index (CFI) and Root Mean Square Error of Approximation (RMSEA) (Hu and Bentler, 1999).

Second, for the predictive and threshold analysis, the analytical dataset was partitioned into a training set (80%, *n* = 409) and a held-out test set (20%, *n* = 103) using stratified random sampling to preserve the distribution of the outcome variable across splits. The test set was strictly withheld from all model development and hyperparameter optimization procedures. A Random Forest regressor was built using the scikit-learn library in Python to predict mental well-being using all measured features and demographic variables (Pedregosa et al., 2011). Feature preprocessing included standardization (zero mean, unit variance) of all continuous predictors. The full feature set entered into the model comprised: Physical Activity (MET-minutes), Sleep Quality (PSQI global score), Cognitive Fatigue (CBI score), Esports Engagement (IGDS9-SF score), weekly esports hours, and demographic covariates (age, gender). Hyperparameter optimization was conducted using nested 5-fold cross-validation on the training set exclusively, with a grid search over the following parameter space: number of trees (n_estimators: 100, 200, 500), maximum features per split (max_features: ‘sqrt’, ‘log2’, 0.5), maximum tree depth (max_depth: None, 10, 20, 30), and minimum samples per leaf (min_samples_leaf: 1, 2, 5). Optimal hyperparameters selected were: n_estimators = 500, max_features = ‘sqrt’, max_depth = 20, min_samples_leaf = 2. Overfitting was evaluated by comparing cross-validated training R^2^ (M = 0.52, SD = 0.04 across folds) with held-out test R^2^ (0.48), indicating minimal overfitting. Model performance is reported with 95% bootstrap confidence intervals computed over 1,000 resamples of the test set (R^2^ = 0.48 [95% CI: 0.38, 0.57]). The SHAP library was employed to interpret the model. SHAP values were computed exclusively on the held-out test set using the TreeExplainer algorithm, ensuring that the presented patterns reflect genuine generalization rather than training data artifacts. SHAP Summary Plots and Dependence Plots were generated to explicitly visualize non-linear trajectories and predictive patterns of physical activity.

## 4 Results

### 4.1 Preliminary analyses and measurement model

Descriptive statistics and bivariate Pearson correlations for the main study variables are comprehensively detailed in Table 1. As shown in Table 1, the correlation matrix provided initial support for the hypothesized relationships. Physical activity exhibited a significant positive correlation with mental well-being and a significant negative correlation with cognitive fatigue. Additionally, reverse-coded sleep quality was strongly associated with reduced cognitive fatigue.

**Table 1:** Descriptive statistics and bivariate correlations.

Variables	Mean	SD	1	2	3	4	5
1. Physical activity (MET-mins)	1850.32	950.45	—				
2. Sleep quality (Reversed)	12.45	3.20	0.38**	—			
3. Cognitive fatigue	48.21	14.50	−0.35**	−0.52**	—		
4. Esports engagement	22.30	6.85	−0.15*	−0.28**	0.44**	—	
5. Mental well-being	14.50	4.60	0.41**	0.46**	−0.58**	−0.32**	—

*N* = 512. *p < 0.05, **p < 0.01. Sleep Quality is reverse-coded so that higher scores represent better sleep.

Prior to structural testing, the measurement model was evaluated. The measurement model demonstrated a robust fit to the data: χ^2^(142) = 285.45, *p* < 0.001, CFI = 0.96, Tucker-Lewis Index (TLI) = 0.95, RMSEA = 0.045, and Standardized Root Mean Square Residual (SRMR) = 0.041. As presented in Table 2, all standardized factor loadings were statistically significant, ranging from 0.68 to 0.92. Composite Reliability (CR) scores for all latent constructs exceeded the recommended threshold of 0.70, and the Average Variance Extracted (AVE) for each construct surpassed 0.50, satisfying the criteria for convergent validity. Discriminant validity was also confirmed for all variables.

**Table 2:** Confirmatory factor analysis and construct validity.

Construct	Items	Factor loadings	CR	AVE
Sleep quality	7	0.70–0.85	0.85	0.54
Cognitive fatigue	13	0.72–0.89	0.90	0.61
Esports engagement	9	0.68–0.83	0.88	0.58
Mental well-being	5	0.78–0.92	0.92	0.68

All factor loadings are standardized and significant at p < 0.001. Physical activity was modeled as an observed variable.

### 4.2 Structural equation modeling and mediation analysis

**Figure 1:**
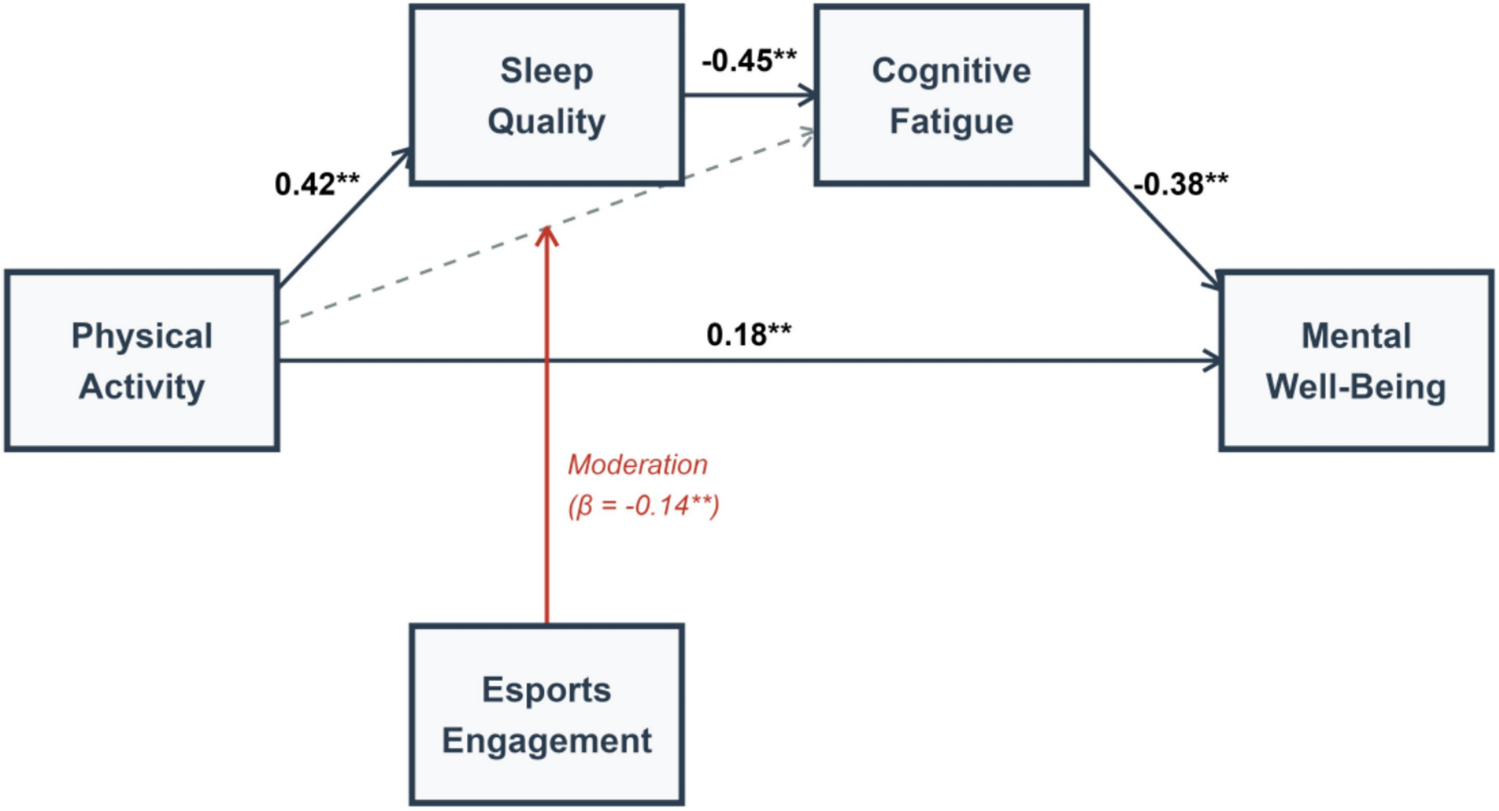
Structural equation model of the mediating pathways. ** indicates *p* < 0.01.

The structural model examining the hypothesized relationships exhibited a strong fit to the data (χ^2^/df = 2.15, CFI = 0.95, RMSEA = 0.048). The theoretical path model, including standardized coefficients, is visually represented in Figure 1. Supporting Hypothesis 1, the direct path from physical activity to mental well-being remained statistically significant after accounting for the mediators.

Mediation analysis utilizing 5,000 bootstrapping iterations supported Hypothesis 2. The detailed unstandardized and standardized path coefficients alongside the bootstrapped confidence intervals are presented in Table 3. As shown in Table 3, the specific indirect effect of physical activity on mental well-being via sleep quality was significant. The specific indirect effect via cognitive fatigue was also significant. Most importantly, the serial indirect effect traversing from physical activity to sleep quality, then to cognitive fatigue, and ultimately to mental well-being was statistically significant. These results indicate that physical activity is associated with the cognitive and sleep-related outcomes inherent in esports, thereby being linked to psychological health in a pattern consistent with the proposed serial mediation pathway.

**Table 3:** Standardized path coefficients and bootstrapped indirect effects.

Pathways	*β*	SE	*p*-value	95% CI (Lower, Upper)
Direct effects				
PA → Sleep Quality	0.42	0.04	<0.001	[0.34, 0.50]
PA → Cognitive Fatigue	−0.20	0.05	<0.001	[−0.30, −0.10]
Sleep Quality → Cognitive Fatigue	−0.45	0.05	<0.001	[−0.55, −0.35]
Sleep Quality → Mental Well-Being	0.30	0.05	<0.001	[0.20, 0.40]
Cognitive Fatigue → Mental Well-Being	−0.38	0.04	<0.001	[−0.46, −0.30]
PA → Mental Well-Being	0.18	0.05	0.002	[0.08, 0.28]
Moderation effect				
Engagement × PA → Cognitive Fatigue	−0.14	0.04	0.001	[−0.22, −0.06]
Indirect effects				
PA → Sleep → Well-Being	0.12	0.02	< 0.001	[0.08, 0.16]
PA → Fatigue → Well-Being	0.08	0.02	0.008	[0.04, 0.12]
PA → Sleep → Fatigue → Well-Being	0.07	0.02	0.002	[0.03, 0.11]

CI, Bias-corrected bootstrap confidence intervals based on 5,000 resamples. PA, Physical Activity.

Regarding Hypothesis 3, moderation analysis revealed that the pathway from physical activity to cognitive fatigue was significantly moderated by esports engagement intensity (*β* = −0.14, *p* < 0.01). Simple slope analysis indicated that for players with high esports engagement, the association between physical activity and cognitive fatigue reduction was substantially stronger compared to those with low engagement. This suggests that highly immersed players may derive greater marginal associational benefits from physical activity.

### 4.3 Explainable machine learning (SHAP) analysis

**Figure 2:**
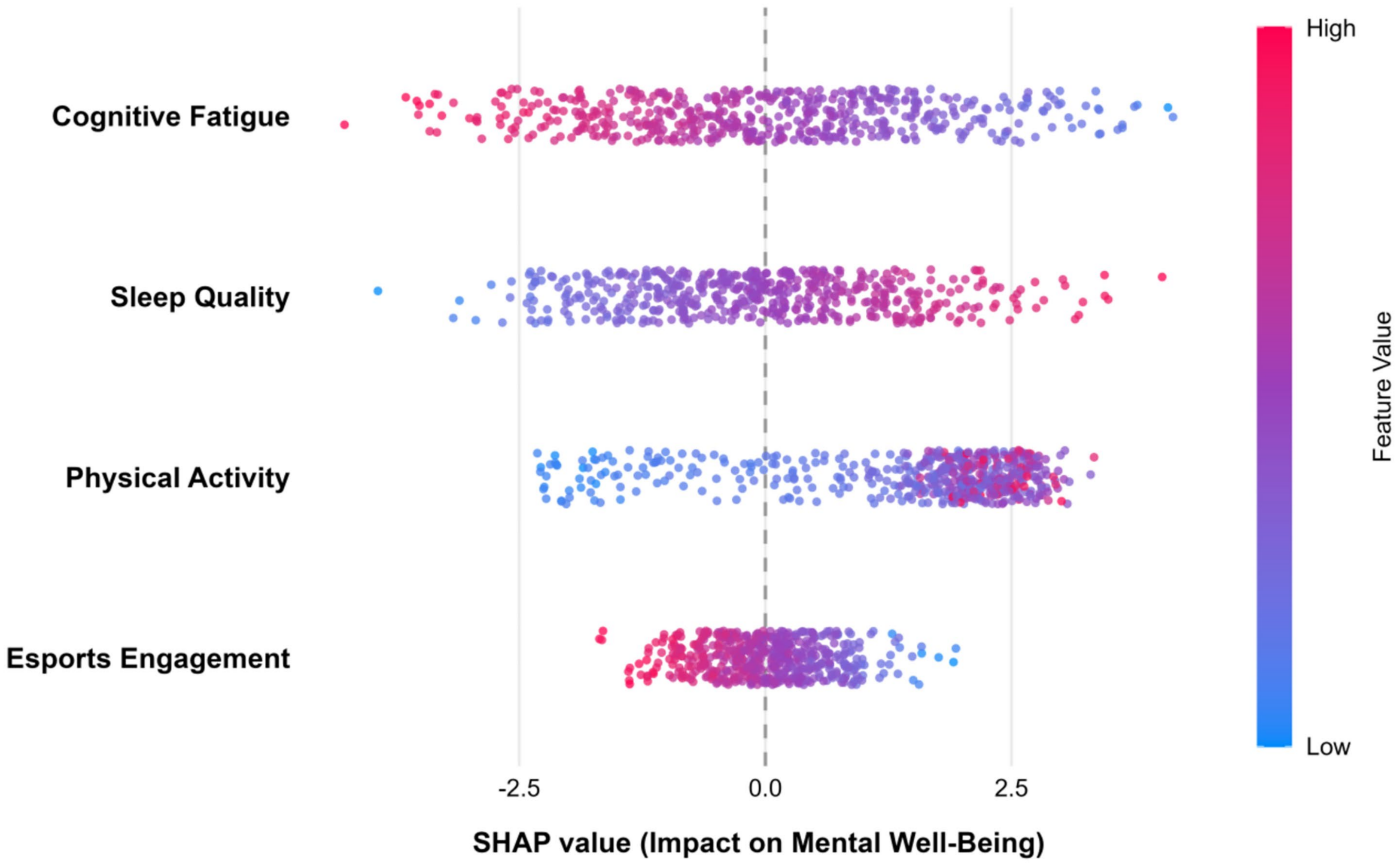
SHAP summary plot for mental well-being prediction.

To capture complex, non-linear dynamics, the Random Forest model was trained to predict WHO-5 scores. The model demonstrated moderate-to-substantial predictive performance on the held-out testing set (R^2^ = 0.48 [95% CI: 0.38, 0.57]). While this value indicates that the model accounts for approximately half of the variance in mental well-being scores, it should be interpreted as reflecting meaningful but not exhaustive predictive capacity, given that unmeasured contextual and individual factors inevitably contribute to well-being outcomes. The global feature importance and directionality are visualized in Figure 2.

As depicted in Figure 2, cognitive fatigue and sleep quality emerged as the top predictors. Physical activity (MET-minutes) ranked as the third most vital predictor of mental well-being, surpassing raw esports engagement hours. In the SHAP summary plot, higher physical activity values are clearly clustered on the positive side of the SHAP value axis, indicating an overall positive predictive association with well-being predictions.

**Figure 3:**
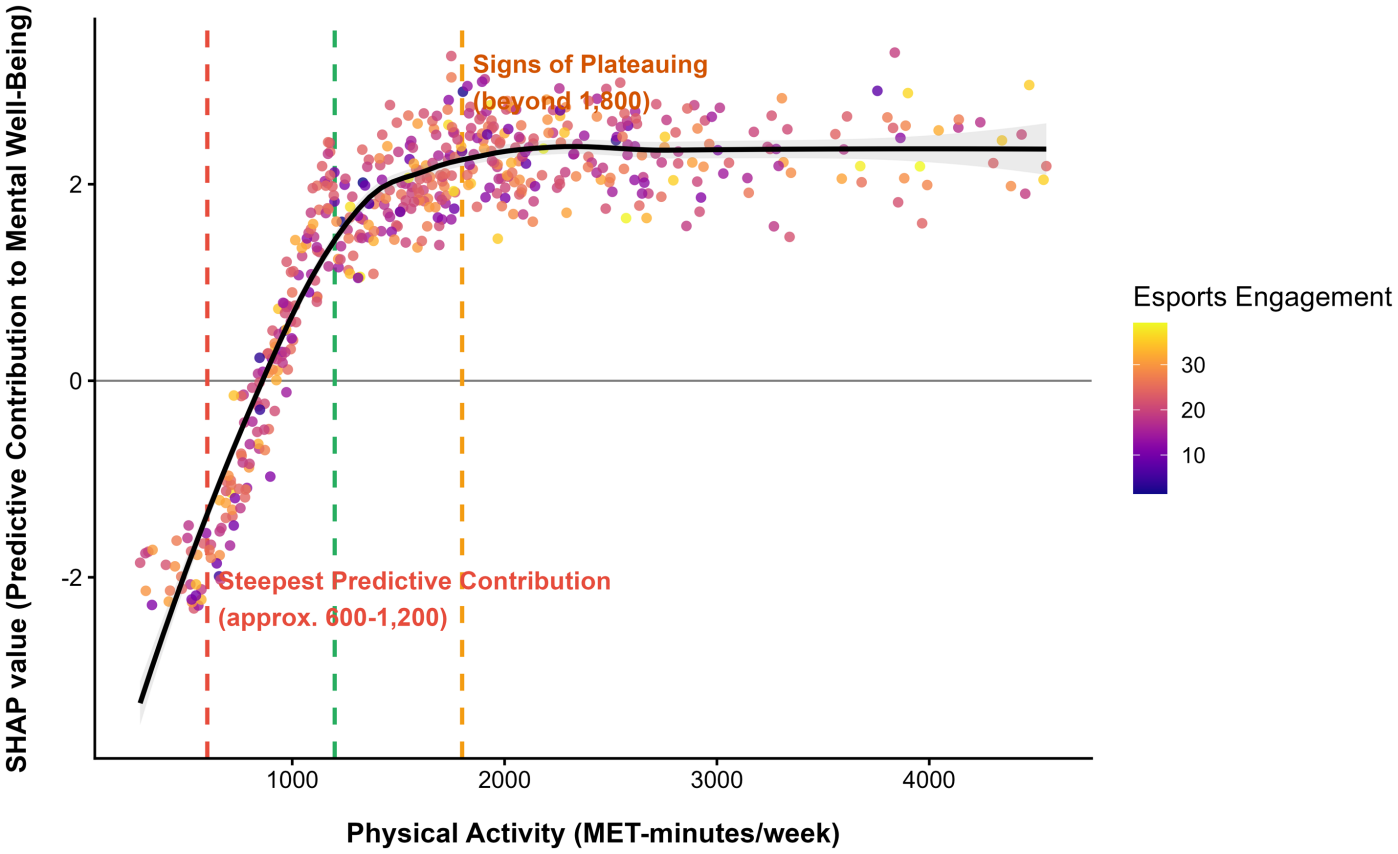
SHAP dependence plot for physical activity.

To address the exploratory behavioral patterns relevant for future intervention research, we analyzed the SHAP Dependence Plot for physical activity, presented in Figure 3.

As shown in Figure 3, the relationship between physical activity and its predicted contribution to mental well-being is distinctly non-linear. The plot reveals a steep, positive trajectory beginning around 600 MET-minutes (approximately equivalent to 150 min of moderate activity per week). The predicted psychological contribution escalates as physical activity increases towards 1,200 MET-minutes. However, beyond approximately 1800 MET-minutes, the curve exhibits a clear plateau, consistent with diminishing marginal returns for mental well-being in this predictive model. It is important to emphasize that these SHAP dependence patterns are exploratory in nature; they reflect predictive associations within the observed data distribution and should not be interpreted as establishing causal predictive patterns or definitive clinical guidelines. Prospective experimental validation is required before these patterns can serve as the basis for clinical or organizational recommendations.

Furthermore, the color gradient in Figure 3 represents the intensity of Esports Engagement. The dispersion pattern of colors across the physical activity spectrum visually corroborates the moderation effect identified in the SEM analysis. Specifically, high-engagement players (yellow/lighter dots) demonstrate a more pronounced variance in SHAP values at lower physical activity levels, suggesting that they may be more sensitive to both the associations between inactivity and reduced well-being, and the predicted benefits of initiating moderate physical activity.

## 5 Discussion

The primary objective of this investigation was to integrate health psychology into the diverse and rapidly expanding context of electronic sports. Utilizing a rigorous dual-analytical approach, this study sought to uncover the mechanisms and predictive patterns through which physical activity is associated with practitioners’ mental well-being. By applying the Biopsychosocial Model through Structural Equation Modeling and expanding the analytical lens via Explainable Machine Learning, this research yields substantial theoretical and practical implications for occupational health in digital arenas.

### 5.1 Theoretical contributions to health psychology

The SEM findings provide robust empirical support for the applicability of the Biopsychosocial framework within the esports domain. The significant serial mediation elucidates a theoretically plausible mechanistic chain: physical activity may function as a fundamental biological regulatory factor associated with enhanced sleep architecture, which is subsequently associated with clearing the intense cognitive fatigue accumulated during competitive gaming. This finding aligns with physiological research suggesting that aerobic exercise promotes slow-wave sleep and modulates cortisol levels, thereby being linked to cognitive restoration (Biddle et al., 2021; Kemp et al., 2021). This contention is further supported by recent empirical evidence demonstrating that physical activity and physical fitness are associated not only with subjective cognitive fatigue, but also with objective cognitive performance gains, including short-term memory improvement (Agostino et al., 2023). This convergent evidence strengthens the theoretical plausibility of the biopsychosocial pathway proposed in the present study, suggesting that the associational benefits of physical activity extend beyond perceived fatigue reduction to encompass measurable enhancements in cognitive capacity directly relevant to esports performance. Consequently, this is consistent with a necessary paradigm shift in digital health research, moving away from predominantly viewing gaming as a psychological addiction, toward recognizing the physiological substrates of digital fatigue. Physical movement appears to be a cornerstone of cognitive resilience, serving as a potential biological foundation upon which psychological well-being may be maintained in sedentary occupations.

Furthermore, the moderation analysis introduces a critical nuance to occupational health psychology. The finding that highly engaged players show augmented associations between PA and well-being outcomes suggests that exercise may act as an active stress-buffering resource. The greater the occupational digital load, the more the biopsychological system may rely on physical exertion to maintain homeostasis and prevent burnout (Trotter et al., 2020). This finding is consistent with the allostatic load theory, suggesting that sedentary behavior among high-performing digital athletes is a structural occupational hazard that may contribute to allostatic overload, potentially requiring targeted behavioral compensation (DiFrancisco-Donoghue et al., 2019).

### 5.2 Methodological innovations via explainable AI (XAI)

A notable methodological advancement of this investigation is the integration of Explainable Artificial Intelligence into health psychology research. Traditional linear models often assume that higher levels of an independent variable consistently yield proportional increases in the dependent variable. However, the machine learning dependency plots generated in this study revealed a sophisticated non-linear reality. The identified pattern—where predicted psychological contributions appear to surge at approximately 150 min of moderate activity and plateau after 300 min—is consistent with the concept of “diminishing returns” in behavioral health strategies.

This observed pattern broadly aligns with the World Health Organization’s general physical activity guidelines (World Health Organization, 2020), and offers preliminary, associational evidence that may inform the discussion of tailoring such guidelines specifically for the sedentary esports phenotype. The SHAP analysis suggests that massive volumes of physical exertion may not be necessary to achieve significant psychological associations; rather, reaching the minimum predicted effective dose may be sufficient to reset the sleep-fatigue cycle. Overtraining, in fact, might add unnecessary physiological stress without yielding additional psychological benefits (Pereira et al., 2022). However, it must be explicitly acknowledged that SHAP dependence plots are exploratory tools that capture predictive associations rather than establishing causal predictive patterns. The apparent precision of the 600–1,200 MET-minute zone represents a data-driven, hypothesis-generating observation to be interrogated in prospective, experimentally controlled research rather than a definitive clinical guideline. This application of SHAP values nonetheless offers a methodological template for future psychological research aiming to extract actionable, precision-based clinical thresholds from complex behavioral datasets.

### 5.3 Practical and clinical implications

From an applied perspective, these preliminary associational findings are potentially relevant for esports organizations, coaches, and sports psychologists seeking to optimize player performance and longevity. The identified predictive patterns represent a preliminary empirical basis that warrants prospective validation prior to clinical implementation, but offer an initial hypothesis-generating framework for informing the design of Just-In-Time Adaptive Interventions in digital contexts. Instead of prescribing generic or overly demanding athletic conditioning regimens—which may increase the risk of physical overtraining or detract from necessary gameplay practice—future evidence-based health strategies informed by this work could be precision-targeted.

Esports teams may consider integrating strategic “micro-exercise breaks” or short bouts of moderate aerobic activity into daily practice schedules to reach the 600–1,200 MET-minute weekly range suggested by the present data. Because late-night screen exposure delays the circadian rhythm and sleep onset (Lee et al., 2021), strategically timing physical activity earlier in the day may maximize homeostatic sleep pressure, thereby potentially mitigating the sleep disruptions commonly reported by professional gamers (Jones et al., 2019). From a translational standpoint, implementing these thresholds in real-world esports settings could leverage existing wearable technology ecosystems. Smartwatches and fitness bands capable of estimating MET-equivalent energy expenditure via accelerometry and heart rate fusion algorithms could provide continuous, passive monitoring of weekly physical activity accumulation. Esports organizations could integrate such devices into standard player health management protocols, with automated alerts triggered when weekly MET-minute accumulation falls below 600 MET-minutes. During intensive training camps, these “micro-exercise breaks”—comprising 10–15 min of moderate aerobic activity (e.g., brisk walking, dynamic stretching routines) scheduled between practice blocks—could serve as a low-disruption behavioral unit. Critically, these breaks need not interrupt competitive scrimmage sessions but could replace transitional idle periods, thus preserving gameplay volume while fulfilling the minimum activity range suggested by the present findings. By incorporating these preliminary data-informed recommendations, the esports industry can proactively address the mental and occupational health challenges threatening player careers.

### 5.4 Limitations and future directions

Despite its methodological rigor, the study is subject to certain limitations that warrant consideration. First, the cross-sectional nature of the survey design precludes definitive causal inferences, and all interpretations throughout this paper should be understood as reflecting predictive associations rather than established causal mechanisms. While the structural models imply directionality based on established theoretical precedents, reciprocal causality—such as higher mental well-being leading to greater physical activity participation—cannot be entirely ruled out. Second, the reliance on self-reported measures for physical activity and sleep, though standard for large-scale psychosocial surveys, may introduce recall and social desirability biases.

A methodological limitation of particular relevance concerns potential common method bias (CMB). All focal constructs—physical activity, sleep quality, cognitive fatigue, esports engagement, and mental well-being—were assessed via self-report within a single survey administration, creating conditions under which CMB may artifactually inflate observed associations (Podsakoff et al., 2003). Several procedural steps were taken to mitigate this risk: survey items for different constructs were separated into distinct sections and randomly ordered within scales, respondent anonymity was guaranteed, and the voluntary nature of participation was emphasized to reduce socially desirable responding. As a post-hoc diagnostic, we conducted Harman’s single-factor test; the largest single factor accounted for 28.4% of the total variance, well below the 50% threshold commonly used as a preliminary indicator of pervasive CMB. While Harman’s test has well-documented limitations as a definitive diagnostic (Podsakoff et al., 2003), this result provides preliminary reassurance that CMB alone is unlikely to fully account for the observed associations. Nonetheless, the possibility that shared method variance inflates some effect sizes cannot be fully excluded. Future studies should incorporate methodological triangulation—specifically, objective accelerometry for physical activity and actigraphy for sleep—to provide source-independent verification of these associations and more definitively rule out CMB as an explanatory factor.

The sample is also predominantly male, which, while reflective of the current professional esports demographic, limits the generalizability of the findings to female gamers. Extending the non-linear dose–response analysis to female esports populations warrants specific theoretical consideration. From a physiological standpoint, sex-based differences in hormonal profiles—particularly estrogen-related modulation of the hypothalamic–pituitary–adrenal axis—may shift the optimal MET-minute thresholds identified here. Estrogen has been shown to have neuroprotective and sleep-regulatory effects, potentially altering the minimum level of PA associated with equivalent cognitive restoration outcomes. From a sociocultural perspective, female esports players may face distinct stressors related to gender minority status within competitive gaming environments, which could interact with PA’s stress-buffering mechanisms differently than observed in the predominantly male sample. Future studies should explicitly recruit sex-balanced samples to test whether the 600–1,200 MET-minute pattern generalizes across genders.

A further limitation concerns the operationalization of esports engagement intensity. The use of the IGDS9-SF as a continuous engagement proxy, while theoretically justified for the present subclinical sample, conflates two conceptually distinct constructs—healthy occupational immersion and problematic gaming symptoms. This constrains the precision of the moderation analysis and the interpretability of the engagement-related findings. Future studies should employ validated engagement-specific instruments to more cleanly capture this dimension.

Finally, the non-linear patterns identified via SHAP dependence plots are exploratory in nature and should not be interpreted as causal dose–response curves. Their apparent precision is conditional on the cross-sectional, self-reported nature of the data and the specific sample from which the Random Forest model was trained. External validation in independent samples—ideally using objective PA monitoring—is necessary before these patterns can inform clinical or organizational practice.

Future research should prioritize longitudinal or quasi-experimental designs to establish causality. Integrating objective wearable telemetry, such as actigraphy for precise sleep architecture monitoring and continuous heart rate monitors for exact physical activity intensity tracking, would cross-validate the machine learning patterns discovered in this study. Additionally, exploring specific modalities of physical activity within esports contexts could provide even more granular intervention guidelines (Herold et al., 2019). It is theoretically plausible that different PA modalities would yield differential benefits for esports practitioners given the distinct physiological demands of their occupation. Aerobic exercise (e.g., running, cycling) is particularly well-positioned to address the sleep-fatigue pathway suggested in this study, as cardiovascular activity promotes slow-wave sleep and elevates brain-derived neurotrophic factor (BDNF) levels, directly supporting the neurobiological mechanism proposed in the biopsychosocial model. By contrast, targeted musculoskeletal exercises—particularly those addressing the upper extremities, cervical spine, and lower back—may predominantly mitigate the musculoskeletal strain component of esports-related physical burden but exert comparatively weaker associations with the sleep architecture and cognitive restoration pathways. Future intervention studies should systematically compare these modalities within esports cohorts to provide exercise prescription specificity.

## 6 Conclusion

As occupational environments become increasingly digital and cognitively demanding, health psychology must continuously adapt its theoretical paradigms to protect these specialized populations. This study demonstrates that physical activity is robustly associated with the preservation of mental well-being in the diverse, sedentary context of esports. Through the innovative integration of structural equation modeling and explainable machine learning, the research moves beyond theoretical linear abstractions to provide associational, hypothesis-generating, threshold-informed insights. Encouraging esports practitioners to integrate targeted physical activity is not a distraction from their digital pursuits, but a direction supported by the present associational data to sustain cognitive excellence and long-term psychological health. These findings should be understood as cross-sectionally based and hypothesis-generating, necessitating prospective experimental validation before they can serve as definitive clinical or organizational guidelines.

## Data Availability

The datasets presented in this article are not readily available due to privacy and ethical restrictions. Requests to access the datasets should be directed to the corresponding author.
